# A Vision-Based Sensor for Noncontact Structural Displacement Measurement

**DOI:** 10.3390/s150716557

**Published:** 2015-07-09

**Authors:** Dongming Feng, Maria Q. Feng, Ekin Ozer, Yoshio Fukuda

**Affiliations:** Department of Civil Engineering and Engineering Mechanics, Columbia University, New York, NY 10027, USA; E-Mails: mfeng@columbia.edu (M.Q.F.); eo2327@columbia.edu (E.O.); yf2290@columbia.edu (Y.F.)

**Keywords:** vision sensor, displacement, template matching, upsampled cross correlation, subpixel resolution, civil engineering structures

## Abstract

Conventional displacement sensors have limitations in practical applications. This paper develops a vision sensor system for remote measurement of structural displacements. An advanced template matching algorithm, referred to as the upsampled cross correlation, is adopted and further developed into a software package for real-time displacement extraction from video images. By simply adjusting the upsampling factor, better subpixel resolution can be easily achieved to improve the measurement accuracy. The performance of the vision sensor is first evaluated through a laboratory shaking table test of a frame structure, in which the displacements at all the floors are measured by using one camera to track either high-contrast artificial targets or low-contrast natural targets on the structural surface such as bolts and nuts. Satisfactory agreements are observed between the displacements measured by the single camera and those measured by high-performance laser displacement sensors. Then field tests are carried out on a railway bridge and a pedestrian bridge, through which the accuracy of the vision sensor in both time and frequency domains is further confirmed in realistic field environments. Significant advantages of the noncontact vision sensor include its low cost, ease of operation, and flexibility to extract structural displacement at any point from a single measurement.

## 1. Introduction

Civil engineering structures including buildings and bridges are exposed to various external loads such as traffic, gust and earthquake during the operational lifetime. Monitoring structural static and dynamic displacements can provide quantitative information for both structural safety evaluations and maintenance purposes. Such practice, however, is highly expensive to operate, mainly due to cumbersome, time-consuming, and expensive installation of sensors and their data acquisition systems. Sensors currently available for measuring structural displacements can be classified as contact-type (e.g., Linear Variable Differential Transformer (LVDT)) and noncontact-type (e.g., GPS, laser vibrometer and radar interferometry system) sensors [1,2,3,4,5,6,7,8]. The contact sensor requires the access to the object structure to install the sensor and physically connect it to a stationary reference point, which is often difficult or even impossible to locate. The GPS sensors are easier to install but the measurement accuracy is limited, usually with errors between 5 and 10 mm [1,2,3,4]. The noncontact laser vibrometer is generally accurate. But the limited measurement distance prevents its applications in monitoring civil engineering structures because longer distance measurement requires the use of a high-intensity laser beam that would endanger human health [4,6]. The interferometric radar system allows remote measurements with a good resolution. However, it requires reflecting surfaces mounted on the structure [5].

To cope with these problems, noncontact vision-based displacement measurement systems have been developed recently, which are primarily enabled by the template matching/registration techniques [1,2,3,9,10,11,12,13,14,15,16,17,18,19]. For example, Busca *et al*. [12] developed a vision-based displacement sensor system using three template matching algorithms, namely, pattern matching, edge detection and digital image correlation (DIC). The vision sensor was used to measure the vertical displacement of a railway bridge by tracking high-contrast target panels fixed to the bridge. Song *et al*. [11] measured the displacement of a cantilever beam from a vision sensor by extracting markers using subpixel Hough transforms from video images. Kim *et al*. [9] proposed a vision-based monitoring system using DIC to evaluate the cable tensile forces of a cable-stayed bridge. Ribeiro *et al*. [3] measured the dynamic displacement of a railway bridge utilizing the RANdom SAmple Consensus (RANSAC) algorithm. On the basis of a robust orientation code matching (OCM) algorithm, the authors developed a vision sensor system for real-time displacement measurement by tracking natural targets on the structural surface, which eliminates the requirement for physical access to structures to install artificial target panels [2,20].

In practice, one major concern for the vision sensor system is the measurement accuracy. Template matching technique usually gives displacement with integer-pixel resolution since the minimal unit in a video image is one pixel. Although in many applications the pixel-level accuracy is adequate, it is often far from the required in case of small structural vibrations. To improve the measurement precision, incorporating the subpixel registration into the template matching algorithm is regarded as the best choice. The interpolation technique is most commonly used subpixel approach, examples of which includes intensity interpolation, correlation coefficient curve-fitting or interpolation, phase correlation interpolation and the geometric methods [21,22,23]. Subpixel registration can also be formulated as an optimization problem and solved through heuristic algorithms such as genetic algorithm, artificial neural network algorithm, and particle swarm optimization, *etc*. [24,25]. There are also other subpixel techniques that are based on Newton-Raphson method [26] and gradient-based methods [27].

As an emerging sensor technique, there is a need to thoroughly investigate the noncontact vison sensor by employing different template matching as well as subpixel algorithms, and experimentally evaluating its performance through tests on various structures. In this study, a novel vision sensor is developed based on an advanced subpixel template matching technique, *i.e.*, the upsampled cross correlation (UCC), which is developed into a software package for real-time displacement extraction. A series of laboratory and field tests are carried out to evaluate its performance.

The paper is organized as follows: in Section 2, the estimation of scaling factor is discussed, and the vision sensor system including the hardware and the theoretical background of the software is introduced; Section 3 evaluates its performance through a laboratory shaking table test of a small-scale frame structure; Section 4 presents two field tests results of a railway bridge and a pedestrian bridge, respectively; Section 5 concludes this study.

## 2. Proposed Vision Sensor System

**Figure 1:**
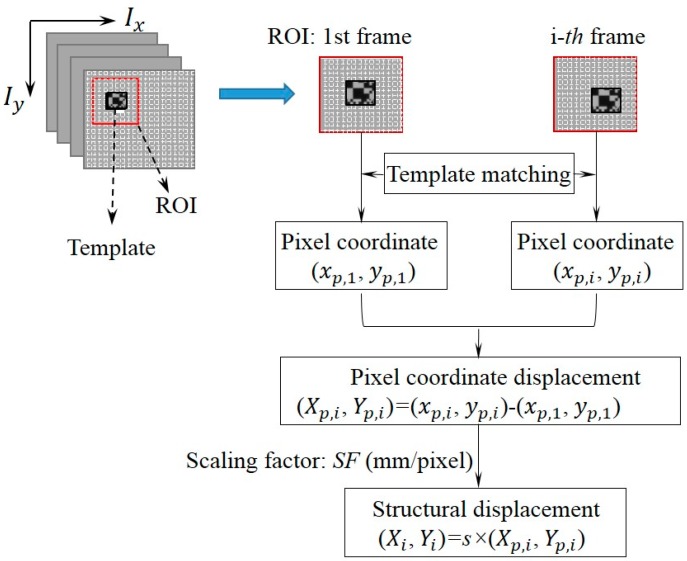
Procedure of vision sensor implementation.

The underlying principle of the vision sensor for displacement measurement is the template matching technique. Figure 1 shows the basic procedure of the vision sensor implementation. In the implementation, an initial area to be tracked is defined as a template in the first image of a sequence of video frames. The template can be located in the successive images using the template matching technique. To reduce computational time, the searching area could be confined to a predefined region of interest (ROI) near the template’s location in the previous image.

### 2.1. Scaling Factor Determination

**Figure 2:**
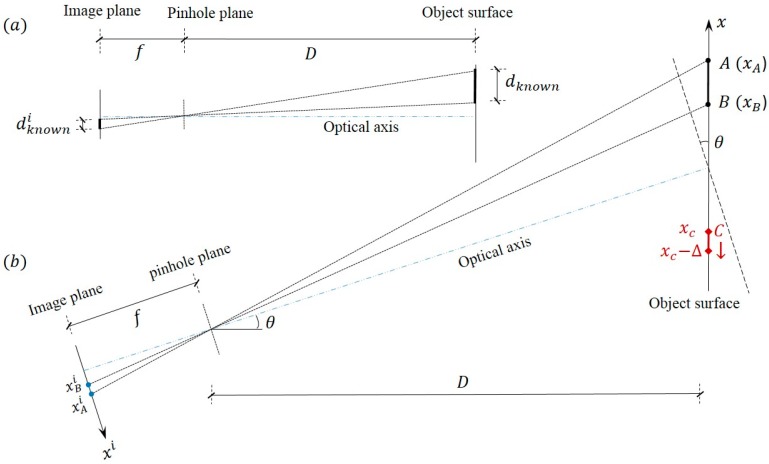
Scaling factor determination: (**a**) optical axis perpendicular to object surface; (**b**) optical axis non-perpendicular to object surface.

In order to obtain structural displacements from the captured video images, the establishment of the relationship between the pixel coordinate and the physical coordinate is required (e.g., with units of mm/pixel). As shown in Figure 2a, when the image plane is parallel to the object surface, the scaling factor in the translational direction (*x* axis) can be determined by:

(1) $$SF=\frac{d_{known}}{I_{known}}\text{ or }SF=\frac{d_{known}}{d_{known}^{i}}d_{pixel}=\frac{D}{f}d_{pixel}$$

where $d_{known}$ is the known physical length on the object surface, $d_{known}^{i}$ and $I_{known}$ are the corresponding physical length and pixel length at the image plane respectively with $d_{known}^{i}=I_{known}d_{pixel}$, $d_{pixel}$ is the pixel size (e.g., in μm/pixel), *D* is the distance between the camera and the object, and *f* is the focal length.

Thus the scaling factor can be obtained from one of the two methods: (1) be estimated from the known physical dimension on the object surface and its corresponding image dimension in pixels (*i.e*., $d_{known}$ and $I_{known}$); (2) be estimated based on the intrinsic parameters of the camera as well as the extrinsic parameters between the camera and the object structure (*i.e*., *D*, *f* and $d_{pixel}$).

However, the prerequisite of Equation (1) is the perpendicularity of the camera’s optical axis to the object surface. Thus all points on the object surface have equal depth of fields [8,28]. Such a requirement would impose some difficulties in the practical implementations because small magnitude of camera misalignment angle can be unnoticed during the experiment setup especially when the object distance from the camera is relatively large. Moreover, in outdoor field tests, it is sometimes unavoidable to tilt the camera optical axis by a small angle in order to track the measured object surface.

Figure 2b shows a schematic when the camera optical axis is tilted about the normal directions of the object surface by an angle θ. Assume line *AB* is known dimension on the object. $x_{A}$ and $x_{B}$ are the coordinates of the two points, and $I_{A}^{i}$ and $I_{B}^{i}$ are the corresponding pixel coordinates at the image plane. The scaling factor can be estimated by:

(2) $$SF_{1}=\frac{x_{A}-x_{B}}{I_{A}^{i}-I_{B}^{i}}$$

From the triangular geometry, $x_{A}$ and $x_{B}$ can be expressed as:

(3) $$x_{A}=\frac{Dx_{A}^{i}}{f\cos^{2}\text{θ}-x_{A}^{i}\cos\text{θ}\sin\text{θ}},x_{B}=\frac{Dx_{B}^{i}}{f\cos^{2}\text{θ}-x_{B}^{i}\cos\text{θ}\sin\text{θ}}$$

where $x_{A}^{i}=I_{A}^{i}d_{pixel}$ and $x_{B}^{i}=I_{B}^{i}d_{pixel}$ are the coordinates at the image plane. When θ is small (sinθ≈0), and $x_{A}^{i}\ll f\text{ and }x_{B}^{i}\ll f$, the scaling factor in Equation (2) can be further estimated and simplified in terms of the intrinsic camera parameters and the extrinsic parameters between the camera and the object structure:

(4) $$SF_{2}=\frac{1}{I_{A}^{i}-I_{B}^{i}}\left(\frac{Dx_{A}^{i}}{f\cos^{2}\text{θ}-x_{A}^{i}\cos\text{θ}\sin\text{θ}}-\frac{Dx_{B}^{i}}{f\cos^{2}\text{θ}-x_{B}^{i}\cos\text{θ}\sin\text{θ}}\right)\approx \frac{D}{f\cos^{2}\text{θ}}d_{pixel}$$

For example, if point *C* in Figure 2b has a small translation Δ along the *x* axis at the object surface, the “true displacement” is:

(5) $$\Delta =x_{C}-(x_{C}-\Delta )=\frac{Dx_{C}^{i}}{f\cos^{2}\text{θ}-x_{C}^{i}\cos\text{θ}\sin\text{θ}}-\frac{Dx_{C}^{i,\Delta }}{f\cos^{2}\text{θ}-x_{C}^{i,\Delta }\cos\text{θ}\sin\text{θ}}$$

where $x_{C}^{i}=I_{C}^{i}d_{pixel}$ and $x_{C}^{i,\Delta }=I_{C}^{i,\Delta }d_{pixel}$ are the coordinates of point *C* before and after translation at the image plane.

**Figure 3:**
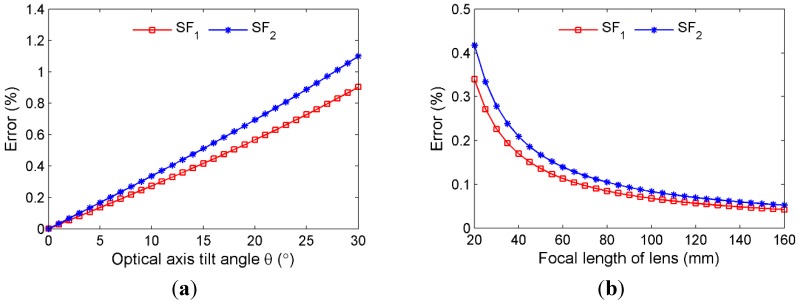
Error resulting from camera non-perpendicularity: (**a**) Effect of optical axis tilt angle (*f* = 50 mm); (**b**) Effect of focal length of lens (*θ* = 3°).

From the scaling factors $SF_{1}$ in Equation (2) and $SF_{2}$ in Equation (4), the “measured displacement” can be estimated by ${\tilde{\Delta }}_{1}=(I_{C}^{i}-I_{C}^{i,\Delta })SF_{1}$ or ${\tilde{\Delta }}_{2}=(I_{C}^{i}-I_{C}^{i,\Delta })SF_{2}$. In order to quantify the error resulting from camera non-perpendicularity, numerical studies are conducted. The measurement errors from the two scaling factors can be defined as: $Error=\left|{\tilde{\Delta }}_{1}-\Delta \right|/\Delta \;\times \;100\%\text{ and }Error=\left|{\tilde{\Delta }}_{2}-\Delta \right|/\Delta \;\times \;100\%$. The adopted parameters are: camera with 640 × 512 pixel resolution, $d_{pixel}=4.8\text{μm}$, $I_{A}^{i}=200$ and $I_{B}^{i}=160$, *D* = 10 m. Point *C* has a 1 pixel translation in the image plane from $I_{C}^{i}=100$ to $I_{C}^{i,\Delta }=99$. The effects of the optical axis tilt angle and lens focal length are investigated by considering a variable range and the results are shown in Figure 3. It can be seen that the error increases as the tilt angle increases and the error is inversely related to the focal length. In sum, it could be concluded that in most practical applications the measurement errors from small optical tilt angles are acceptable. Although this study is based on the 1D (*x* axis) in-plane translation, the conclusions can be equally extended to the 2D in-plane translation.

It is also found from the numerical study that for a fixed camera setup, the measurement error from scaling factor $SF_{1}$ would decrease when the measurement point *C* gets closer to the known dimension *AB*. Especially, the error is minimized when the measurement point is located within the region of known dimension. For scaling factor $SF_{2}$ in Equation (4), errors would further arise from the uncertainties in the tilt angle estimation, camera distance measurement and focal length readings from the adjustable-focal-length lens.

In the laboratory and field tests of this study, scaling factor *SF*_1_ is adopted, which is obtained from Equation (2) based on the known physical dimension on the object surface (e.g., the size of artificial target panels or the size of the nuts and rivets known from the design drawings) and the corresponding image dimension in pixels. It is noted that camera calibration according to Zhang method [29] would reduce the effect of lens distortion [19,30], which is however not carried out in this study.

### 2.2. Hardware of the Vision Sensor System

As tabulated in Table 1, the proposed vision sensor system simply consists of a video camera, a zoom lens and a notebook laptop. During the test, the camera equipped with the lens is fixed on a tripod and placed at a remote location away from the structure. The camera is connected to a laptop installed with the real-time image-processing software. It is noteworthy that setting up the vision sensor, including focusing the lens on the targets, takes only a few minutes.

**Table 1:** Technical specifications of the proposed vision sensor system.

Component	Model	Technical Specifications
Video camera	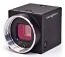 Point Grey/FL3-U3-13Y3M-C	Maximum resolution: 1280 × 1024
		Frame rate: 150 FPS
		Chroma: Mono
		Sensor type: CMOS
		Pixel size: 4.8 μm
		Lens mount: C-mount
		Interface: USB3.0
Optical lens	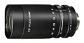 Kowa/LMVZ990 IR	Focal length: 9 to 90 mm
		Maximum Aperture: F1.8
		Mount: C-mount
Laptop computer	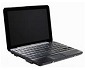 Sony /PCG-41216L	Intel(R) Core(TM) i7-2620M CPU @ 2.70 GHz
		8192 RAM
		250 HDD
		14.1" Screen
Tripod and Accessories	Tripod, USB3.0 type-A to micro-B cable, *etc*.	

### 2.3. Upsampled Cross Correlation for Template Matching

In this study, the vision sensor is developed based on UCC, a subpixel template matching method proposed by Guizar-Sicairos *et al*. [31]. Consider a pair of images f(x,y) and t(x,y) with identical dimensions M×N, among which, t(x,y) has a relative translation from the reference image f(x,y). The cross correlation between f(x,y) and t(x,y) by means of Fourier transform can be defined as:

(6) $$R_{FT}(x_{0},y_{0})=\sum_{x,y}f(x,y)t^{*}(x-x_{0},y-y_{0})=\sum_{u,v}F(u,v)T^{*}(u,v)exp[i2\pi \left(\frac{ux_{0}}{M}+\frac{uy_{0}}{N}\right)]$$

where the summations are taken over all image points (x,y); $\left(x_{0},y_{0}\right))$ is an amount of coordinate shift; “*” denotes complex conjugation; F(u,v) and $T^{*}(u,v)$ represent the discrete Fourier transform (DFT) of their lowercase counterparts, for example

(7) $$F(u,v)=\sum_{x,y}\frac{f(x,y)}{\sqrt{MN}}exp\left[-i2\pi \left(\frac{ux}{M}+\frac{uy}{N}\right)\right]$$

From Equation (6), an initial displacement estimation with pixel-level resolution can be easily acquired by locating the peak of $R_{FT}$. Subsequently, cross correlation based on a time-efficient matrix-multiplication discrete Fourier transform (DFT) is performed in a neighborhood around the initial peak to achieve a subpixel resolution.

**Figure 4:**
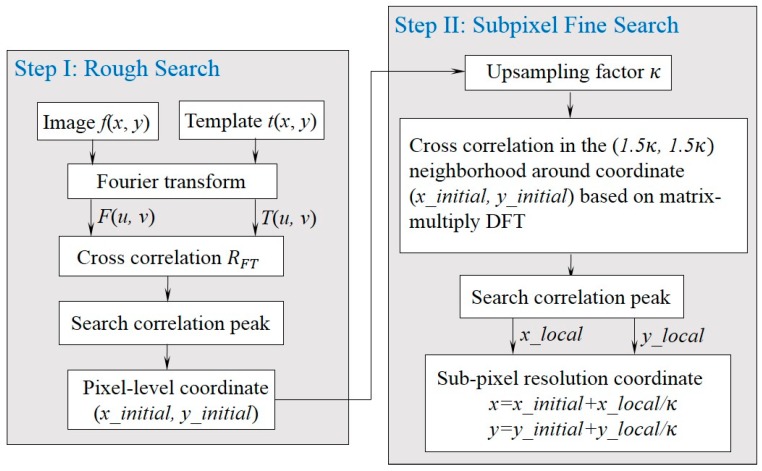
Flowchart of the upsampled cross correlation (UCC) implementation.

Figure 4 shows the flowchart of the vision sensor based on the subpixel UCC, described as follows:

*Step I*: Pixel-level rough search. Compute the cross correlation between the image to register and the reference image by means of Fourier transform, and the initial displacement can be estimated from the correlation peak;

*Step II*: Subpixel fine search. Compute the cross correlation in a 1.5 × 1.5 pixel neighborhood around the initial estimate by an upsampling factor of κ. Thus a subpixel resolution within 1/κ of a pixel is achieved by searching the peak in this (1.5κ,1.5κ) neighborhood. For example, by setting κ=10, a 0.1 subpixel accuracy can be achieved.

In Step II, instead of computing a zero-padded FFT, a matrix-multiplication DFT operation is implemented by the product of three matrices with dimensions (1.5κ,N), (N,M) and (M,1.5κ). The algorithm complexity for this upsampling subpixel search is O(MNκ), while complexity of conventional FFT upsampled by zero-padding $F(u,v)T^{*}(u,v)$ is $O(MN\text{κ}\left[\log_{2}(\text{κ}M)+\text{κ}\log_{2}(\text{κ}N)\right])$. The substantial improvement dramatically reduces computational time and memory requirement without sacrificing accuracy, making possible of real-time displacement measurement.

A real-time video-processing software is developed based on UCC. The programming environment for the software package is Visual Studio 2010 using C++ language. During measurement, the FlyCapture Software Development Kit (SDK) by Point Grey Research is used to capture video images from Point Grey USB 3.0 cameras using the same application programming interface (API) under 32- or 64-bit Windows 7/8 operating system. Then the frame-by-frame image are processed by the UCC algorithm and displayed on the screen using DirectShow library. Meanwhile, the measured displacement history would be shown on the screen in real time and saved to the computer. The online measurement avoids the time-consuming and memory-intensive task of saving huge video files. However, a tradeoff among measurement points, video resolution, maximum frame rate per second and template sizes is necessary. On the other hand, the developed software can also be used for post-processing the recorded video files, which enables the flexibility to extract structural displacements at more points from a single recording.

## 3. Shaking Table Test of a Frame Structure

**Figure 5:**
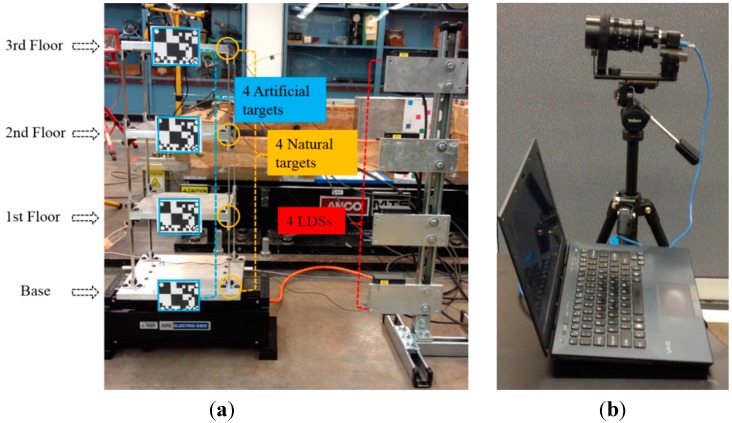
Laboratory test: (**a**) Shaking table test setup; (**b**) Vision sensor system setup.

The performance of the proposed vision sensor is first evaluated through a shaking table test of a scaled three-story frame structure in the Carleton Laboratory at Columbia University, as shown in Figure 5. The aluminum frame structure is bolt-connected for all the column-floor connections.

### 3.1. Shaking Table Test Setup

During the testing, the shaking table (Model# APS113 by APS Dynamics Inc.: San Juan Capistrano, CA, USA) is driven by white noise signals. Four predesigned black and white artificial targets (99 mm × 75 mm) are mounted on the structure for motion tracking. Meanwhile, four bolt connections are used to study the performance of the vision sensor to track natural targets on the structure. As references, the displacements are also measured by four high-accuracy laser displacement sensors or LDSs (Model#LK-G407 by KEYENCE), which are installed between each floor of the frame model and stationary reference points.

The visions senor system is placed 8 m away from the shaking table. During the measurement, video images captured by the camera are digitized into 640 × 512 pixel images in 8 bit grey scales and streamed into the computer through an USB 3.0 cable. Before testing, the processing time for each video frame by the developed software should be obtained to determine the maximum frame rate. In this test, four small areas from the artificial targets and four bolt-connection areas at all floors are simultaneously registered as templates. It is observed that a total time of 5.6 ms is needed for each video frame, including the reading and preparing, template matching and image displaying time. Thus, real-time displacement time histories at eight measurement points can be simultaneously measured with a sampling rate of 150 fps.

### 3.2. Subpixel Resolution Performance

Pixel-level template matching may result in unacceptable measurement errors if the displacement to be measured has same order of magnitude as the scaling factor. In this case, the subpixel technique should be adopted to make template matching fall at a fractional pixel location. To better understand how the subpixel technique improves the measurement precision, displacements extracted from video images by tracking the artificial target on the base floor are used as a demonstration. Four subpixel levels, namely, levels of one integer pixel, 0.5 pixel, 0.2 pixel and 0.05 pixel are chosen, with the corresponding resolutions tabulated in Table 2. Recall again, a desired subpixel resolution can be easily achieved by simply adjusting the upsampling factor κ. In this testing, the scaling factor is 1.338 mm/pixel, providing ±0.669 mm resolution.

**Table 2:** Different levels of subpixel resolution.

**Subpixel (pixel)**	1	0.5	0.2	0.05
**Resolution (mm)**	±0.669	±0.335	±0.134	±0.034

**Figure 6:**
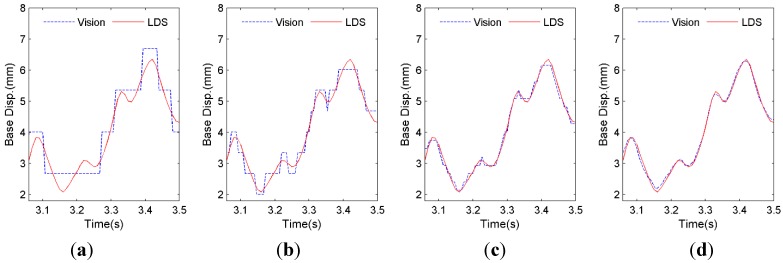
Subpixel resolution evaluation (**a**) Resolution: ±1.338 mm; (**b**) Resolution: ±0.669 mm; (**c**) Resolution: ±0.268 mm; (**d**) Resolution: ±0.067 mm.

As shown in Figure 6a, for the integer-pixel resolution (1.338 mm), the displacement errors between the vision sensor and LDS can be observed clearly. On the other hand, after employing different levels of subpixel analysis, the displacement by the vision sensor agrees better with that by LDS as the resolution improves (with NRMSE errors (by Equation (8)) of 6.41%, 3.80%, 1.73%, and 1.35% respectively for the four zoom-in segments in Figure 6).

It is noted that in the ideal case where video images have no distortion or noise, larger upsampling factor κ would yield smaller error. However, the subpixel accuracies reported in many studies vary within orders of magnitude from 0.5 to 0.01 pixel [21,32], as images may be contaminated by various external environmental noises and system noises arising from the electronics of the imaging digitizer. For the following tests in this study, κ=20 is selected.

### 3.3. Measurement Evaluation by Tracking both Artificial and Natural Targets

To evaluate the performance of the vision sensor, displacements are measured by tracking both the black and white artificial targets and natural targets (*i.e*., bolt connections) and compared with those by four LDSs. Here, the measurements are termed as Vision (artificial target), Vision (natural target) and laser displacement sensor (LDS), respectively.

**Figure 7:**
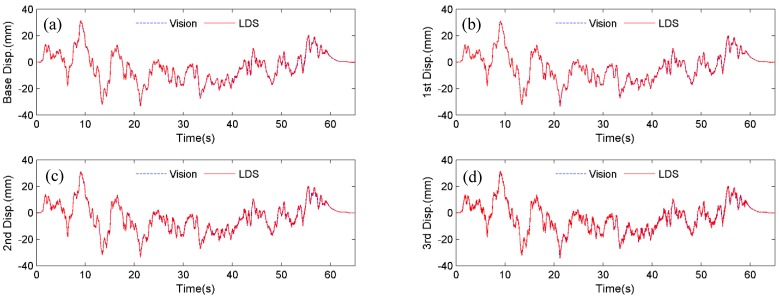
Displacement comparisons between Vision (artificial target) and LDS: (**a**) Base floor; (**b**) 1st floor; (**c**) 2nd floor; (**d**) 3rd floor.

**Figure 8:**
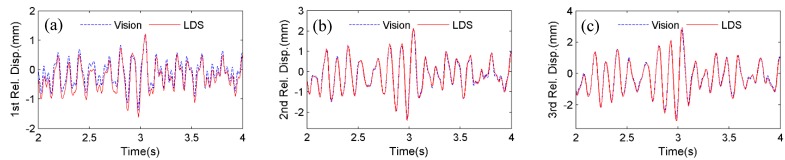
Comparisons of displacement relative to base floor between Vision (artificial target) and LDS: (**a**) 1st floor; (**b**) 2nd floor; (**c**) 3rd floor.

Firstly, the four artificial targets in Figure 5 are used as the tracking target for the vision sensor. Figure 7 shows the comparison of displacements by Vision (artificial target) and LDS. Excellent agreements can be observed. In Figure 8, the plotted displacements of 1st, 2nd, and 3rd floor are relative to the base-floor displacement in Figure 7a, and only enlarged time segments between 2 s and 4 s are shown for better illustration. As shown in Figure 8a, small discrepancies are observed in the 1st floor relative displacements by the vision sensor and LDS. However, considering the small vibration amplitude (smaller than 1 mm), the errors are acceptable. As can be seen from the relative displacements of 2nd and 3rd floor in Figure 8b,c, the discrepancies are reduced as the vibration amplitude increases. And it is expected that the errors would further decrease as the amplitude of the relative vibration increases.

It is noted that the scaling factor for the vision sensor in the testing is 1.338 mm/pixel, meaning that the expected maximum error is 0.669 mm from a pixel-level template matching. However, since an upsampling factor of κ=20 is selected to achieve a 0.05 subpixel accuracy, the vision sensor can still accurately capture the small relative displacements ranging from 0 to 3 mm.

**Figure 9:**
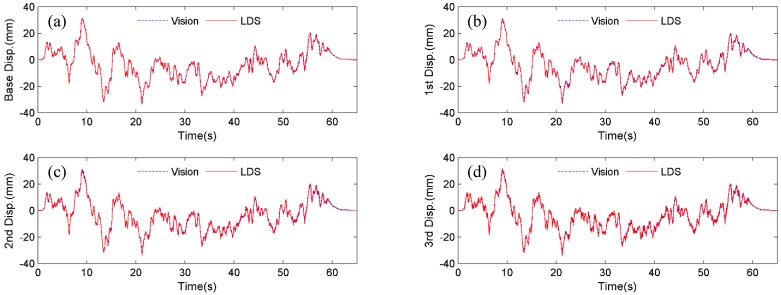
Displacement comparisons between Vision (natural target) and LDS: (**a**) Base floor; (**b**) 1st floor; (**c**) 2nd floor; (**d**) 3rd floor.

**Figure 10:**
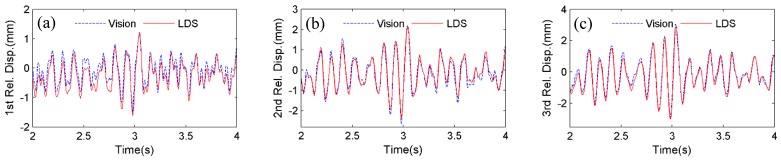
Comparisons of displacement relative to base floor between Vision (natural target) and LDS: (**a**) 1st floor; (**b**) 2nd floor; (**c**) 3rd floor.

Next, instead of tracking the artificial targets, the four bolt connections on the frame structure in Figure 5, are used as the tracking targets. Figure 9 shows the displacement comparison of each floor by Vision (natural target) and LDS, respectively. Figure 10 plots the 1st, 2nd, and 3rd floor displacements relative to the base floor. Again, satisfactory agreements between Vision (natural target) and LDS can be achieved.

To quantify the accuracy and precision of the vision sensor, error analysis is performed using the normalized root mean squared error (NRMSE)

(8) $$\text{NRMSE=}\frac{\sqrt{\frac{1}{n}\sum_{i=1}^{n}{\left(x_{i}-y_{i}\right)}^{2}}}{y_{\max}-y_{\min}}\times 100\%$$

where *n* = number of measurement data; $x_{i}$ and $y_{i}=i\text{th}$ displacement data at time $t_{i}$, measured by the vision sensor and the LDS, respectively; and $y_{\max}=\max(y_{i}),\;y_{\min}=\min(y_{i})$.

Table 3 tabulates the NRMSE errors of the displacement measurements in Figure 7 and Figure 9. It is concluded that vision sensor demonstrates a high accuracy by tracking either artificial targets or natural targets, with a maximum NRMSE error of 0.6%. By tracking natural targets without requiring artificial targets installed on fixed locations on the structure, the vision sensor provides the flexibility to easily change locations for displacement measurement, thus further facilitating the testing process.

**Table 3:** Measurement errors: normalized root mean squared error (NRMSE) (%).

Floor	Vision Sensor	
	Artificial Target	Natural Target
Base	0.39	0.60
1st	0.28	0.45
2nd	0.27	0.35
3rd	0.18	0.32

## 4. Field Tests

To evaluate the performance of the vision sensor in realistic field environments, field tests are carried out on two bridges. Specifically, the time-domain performance is evaluated through field tests of a railway bridge by comparing with reference LVDT, and the frequency-domain performance is evaluated through field tests of a pedestrian bridge by comparing with the conventional accelerometer. A sampling rate of 150 frames per second with a resolution of 640 × 512 pixel was adopted.

### 4.1. Field Test of a Railway Bridge

**Figure 11:**
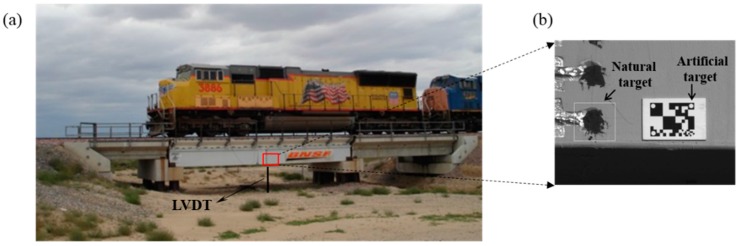
Field test of a railway bridge: (**a**) Displacement measurement under moving trainloads; (**b**) Artificial target and natural target.

In collaboration with the Transportation Technology Center, Inc. (TTCI), field measurements are carried out on a state-of-the-art hybrid composite bridge, which is one of the test-bed bridges in TTCI, Colorado. As shown in Figure 11a, the bridge is 12.8 m long. The train used for the testing has one locomotive and 15 freight cars. Figure 11b shows the artificial target and natural target on the bridge. The video camera is fixed on a tripod and set up at a remote location away from the bridge. This field tests focused on the measurement of the vertical displacement at the mid-span point by the vision sensor. As a reference sensor, a conventional contact-type displacement sensor, *i.e*., a LVDT, is installed on the mid span of the bridge with one end connected to a stationary reference point on the ground through a string.

Often in the field, it is difficult to find a location close to the structure to set up the vision sensor system, thus requiring to evaluate performance of vision sensor at different remote distances. Table 4 summarizes two of the field testing cases. Test T1 is conducted with a train speed of 40.23 km/h (or 25 mph) and with the vision sensor system placed 30.48 m (or 100 ft) away from the bridge, and Test T2 with a train speed of 64.36 km/h (or 40 mph) and with the sensor 60.96 m (or 200 ft) away from the bridge. It is noteworthy that the camera optical axis is tilted by small angles (2° for T1 and 1° for T2) with respect to the normal direction of the bridge surface, the errors from which are acceptable based on the scaling factor discussions in Section 2.1.

**Table 4:** Test cases.

Test	Measurement Distance (m)	Camera Tilt Angle (°)	Train Speed (km/h)	Scaling Factor (mm/pixel)
T1	30.48	2	40.23	1.90
T2	60.96	1	64.36	3.83

**Figure 12:**
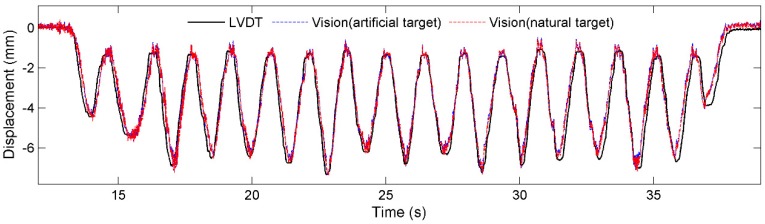
Comparison of displacements: Test T1.

**Figure 13:**
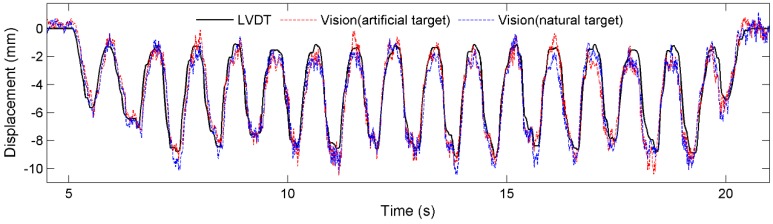
Comparison of displacements: Test T2.

Figure 12 and Figure 13 plot respectively the displacement time histories for tests T1 and T2 by the three sensor systems, namely, the LVDT, Vision (artificial target), and Vision (natural target). In general, the measurements agree well with one another. Specifically, the test results show that the measurement error increases as the measurement distance increases, mainly caused the increased difficulty in tracking either the artificial target or the natural target as measurement distance increases.

During the field tests, two other problems are recognized, which would also contribute to the measurement errors. Firstly, the camera vibrations caused by moving-train-induced ground motion can affect the measurement accuracy when the camera is placed far away from the measurement target and zoom lenses magnifies not only the images but also the camera vibration. This problem becomes more serious for the proposed compact and portable vision-based displacement sensor system, because it is impossible to utilize stable concrete camera base fixture to avoid the micro camera vibration. The second problem is the heat haze that occurs when the air is heated, non-uniformly, by the high ambient temperature during the field testing. The non-uniformly heated air causes variation in its optical reflection index, resulting in image distortion, which would cause more measurement errors as the measurement distance increases, because the air thickness between the target object and the lens of the camera becomes large.

### 4.2. Field Test of a Pedestrian Bridge

**Figure 14:**
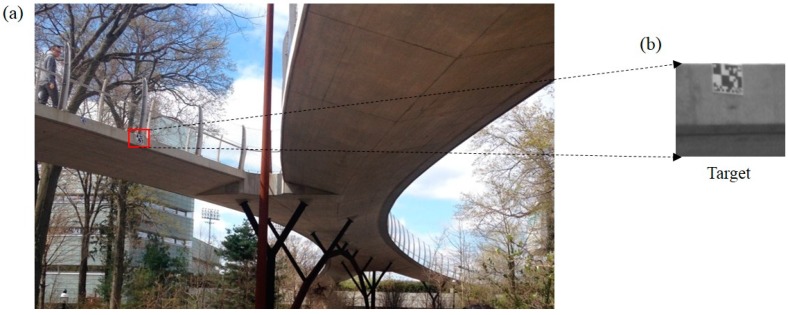
Field test: (**a**) Streicker Bridge; (**b**) Artificial target.

The Streicker Bridge is a pedestrian bridge located on the Princeton University campus, NJ, USA. The bridge has a main span and four approaching legs. The main span is a deck-stiffened arch and the legs are curved continuous girders supported by steel columns [33]. This field tests are to study the performance of the vision sensor in frequency domain. Two sets of dynamic loading tests are carried out on the third span of the southeast leg. As shown in Figure 14, one artificial target and one accelerometer (Model#W352C67 by PCB PIEZOTRONICS Inc.: Depew, NY, USA) are installed on the mid span. It is noted that the camera optical axis is tilted by an approximate angle of 15° with respect to the normal direction of the bridge surface, However, in this field test, due to the large height between ground and the bridge bottom surface, it is very difficult to install a reference LVDT to compare the accuracy of the measured displacement time histories by the vision sensor.

**Figure 15:**
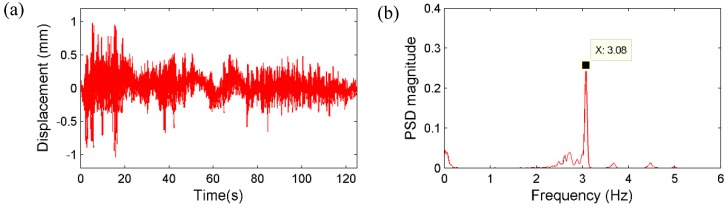
Randomly running of pedestrians: (**a**) Displacement by the vision sensor; (**b**) corresponding PSD.

**Figure 16:**
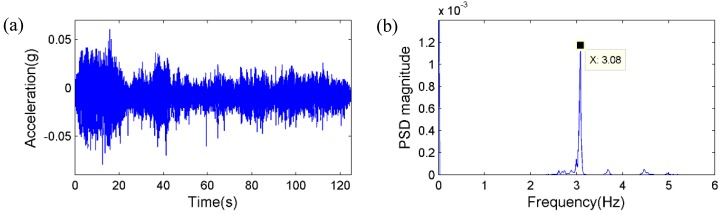
Randomly running of pedestrians: (**a**) Acceleration measurement; (**b**) Corresponding PSD.

First, in order to apply dynamic loads with broadband frequency contents to the bridge, a group of pedestrians ran on the bridge deck randomly with different, varying speeds, rhythms and directions without any particular pattern. Figure 15 shows the displacement measurement from the vision sensor together with the power spectral density (PSD) result. Figure 16 plots the acceleration measurement from the accelerometer and the corresponding PSD result. By comparing the results, one dominant frequency can be clearly identified as 3.08 Hz from both sensors, as well as two higher frequencies of 3.68 Hz and 4.47 Hz, respectively.

**Figure 17:**
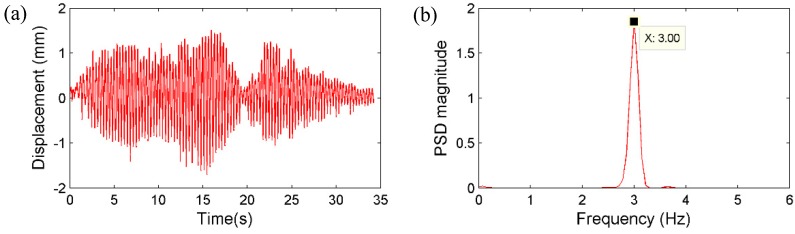
Jumping of pedestrians: (**a**) Displacement by the vision sensor; (**b**) Corresponding PSD.

**Figure 18:**
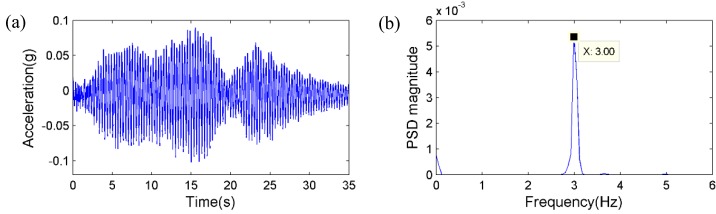
Jumping of pedestrians: (**a**) Acceleration measurement; (**b**) Corresponding PSD.

Secondly, the pedestrian participants jumped on the mid span of the bridge deck synchronically with a frequency of around 3 Hz, which is close to the estimated first natural frequency of the bridge. Figure 17 and Figure 18 plot the displacement and acceleration time histories obtained respectively from the vision sensor and the accelerometer, together with corresponding PSD results. Again, the identified frequencies based on the two sensors show excellent agreement. Therefore, it is concluded that the same frequency components can be accurately obtained from the vision sensor.

## 5. Conclusions and Future Work

In this study, a vision sensor system is developed for remote measurement of structural displacements based on an advanced subpixel template matching technique, namely, the upsampled cross correlation by means of Fourier transform. Comprehensive experiments, including a shaking table test and two bridge field tests, are carried out to investigate its performance. The following conclusions can be drawn:

(1) As a significant advantage of the proposed vision sensor, better subpixel resolution can be easily achieved by adjusting the upsampling factor. Thus structural vibrations smaller than 1 mm can be accurately measured.
(2) From the shaking table test of a frame structure, satisfactory agreements are observed between the multi-point displacement time histories measured at all floors by one camera by tracking bolt connections on the structure surface and those by four laser displacement sensors.
(3) In realistic field environments, the time-domain performance of the vision sensor is further confirmed through field tests of a railway bridge during train passing; and the frequency-domain performance is validated through field tests of a pedestrian bridge subjected to dynamic loading.

By tracking existing natural targets on the structure surface, the vision sensor developed in this study provides the flexibility to easily change locations for displacement measurement. The availability of such as a remote sensor will facilitate cost-effective monitoring of civil engineering structures.

As part of our plan to improve the measurement accuracy in uncontrolled outdoor field environments, we are working to reduce the errors caused by the heat haze and camera vibrations. Moreover, in order to study the potentials of the vision sensor for structural health monitoring, we are building simply beam specimens with different kinds of damages. The aim is to evaluate the effectiveness of the vision sensor with respect to: (1) measuring full-field displacement responses using one camera; and (2) extracting modal information (natural frequencies and mode shapes) from measured multi-point displacements and detecting damages in beam structures.
